# Radiological presentation of patients of pulmonary tuberculosis with diabetes mellitus

**DOI:** 10.4103/0970-2113.76308

**Published:** 2011

**Authors:** Anand K. Patel, Kiran C. Rami, Feroz D. Ghanchi

**Affiliations:** *Department of Pulmonary Medicine, S. B. K. S. Medical Institute & Research Centre, Sumandeep Vidhyapeeth, Piparia, Vadodara, India. E-mail: dranandkpatel@gmail.com*; 1 *Govt. Medical College, S. S. G. Hospital, Vadodara, India*; 2 *Shree M. P. Shah Medical College, Guru Gobindsing Hospital, Jamnagar, Gujarat, India*

Diabetic patients are considered as a high-risk population for the development of pulmonary tuberculosis (PTB). Usually, PTB is found predominantly in the upper lobes. Lower lung field tuberculosis occurs but is often misdiagnosed as pneumonia, carcinoma, or lung abscesses. In a number of published comparative studies, chest X-ray images from patients having PTB with diabetes mellitus (DM) have been described as ‘atypical’, mainly because they frequently involve the lower lung fields, often with cavities.[1,2] A higher frequency of multi-lobar involvement has also been described among PTB with DM patients.[1] However, other authors have been unable to find differences in the chest X-ray patterns of pulmonary tuberculosis in diabetic and non-diabetic patients.[3] Thus, whether diabetic subjects present atypical radiological presentation of pulmonary TB is still controversial.

We studied 50 patients with pulmonary tuberculosis with or without extra pulmonary tuberculosis having DM. All other forms of extra pulmonary tuberculosis, and HIV seropositive patients were excluded from the study to allow better data comparison. All were subjected to sputum smear for AFB examination, X-ray chest PA view and hematological investigations. Bronchoalveolar lavage and mantoux test were performed only in selected patients. Patients were considered to have a diagnosis of diabetes mellitus if they were receiving insulin or an oral hypoglycemic agent at the time of hospital admission or were found to have two or more fasting blood glucose levels greater than 140 mg%. Upper lung field tuberculosis was defined as tuberculosis involving upper zone. Lower lung field tuberculosis was defined as tuberculosis involving the middle zone and/or lower zone. Cavitation was considered to be present only when its diameter was more than 2 cm.

We found that there was a higher involvement of lower lung field (84%) as compared to upper lung field (16%). Bilateral involvement was present in 32% while unilateral involvement was present in 68%. Ten patients out of the 50 had cavitary disease. Cavitary lesions were more frequently confined to lower lung field (80%). Nodular lesions were found in 36%, exudative lesions were found in 22% and mixed lesions were found in 22%.

There had been much debate concerning the atypical radiographic findings of TB. Some authors[3] have reported no major differences while others[4] have reported a higher involvement of the lower lung fields. Our study supports that tuberculosis tends to occur predominantly at the lower lung fields in patients with diabetes. Some of the previous studies have reported cavitary lesions to be more common among diabetic patients.[2] Some studies[1] have reported higher frequency of multiple cavities among diabetic patients. But this was not observed in the current study. Reasons for atypical radiological images in tuberculosis patients with associated diabetes are not clear.

We conclude that the patients with tuberculosis and DM are more likely to present with atypical radiological images. Among diabetic patients presenting with lower lung field lesions, possibility of TB should always be considered for prompt diagnosis and management.
